# Complete mitochondrial genome sequence of *Pulveroboletus ravenelii* (Boletales, Basidiomycota)

**DOI:** 10.1080/23802359.2022.2110006

**Published:** 2022-09-02

**Authors:** Sung-Eun Cho, Young-Nam Kwag, Sang-Kuk Han, Dong-Hyeon Lee, Chang Sun Kim

**Affiliations:** a Forest Biodiversity Division, Korea National Arboretum, Pocheon, South Korea; b Division of Forest Insect Pests and Diseases, National Institute of Forest Science, Seoul, South Korea

**Keywords:** Basidiomycota, *Boletaceae*, mitochondrial genome, phylogenetic analysis

## Abstract

*Pulveroboletus ravenelii*, an ectomycorrhizal fungus, has long been used in traditional Chinese medicine and mushroom dyes; however, its mitogenome and phylogenetic relationship with other taxa remain unexplored. Here, we sequenced the mitochondrial genome of *P. ravenelii* using next-generation sequencing and found that its mitogenome, a circular DNA molecule of 43,528 bp, comprised 15 protein-coding genes, 15 transfer RNA genes, and two ribosomal RNA genes. The mitogenome had a base composition of A (37.52%), C (11.14%), G (12.21%), and T (39.13%) and a GC content of 23.35%. A phylogenetic tree was constructed to determine the phylogenetic relationship of this species with other taxa based on the mitochondrial genome sequence. This study revealed the phylogenetic positions of *P. ravenelii* and its related genera for the first time.

Mushrooms in the family *Boletaceae* (Basidiomycota) are ecologically and economically important. Recently, seven subfamily-level major clades were identified in *Boletaceae*, and the *Pulveroboletus* group was discovered as a new clade (Wu et al. 2014). *Pulveroboletus ravenelii* (Berk. & M.A. Curtis) Murrill 1909, commonly known as Ravenel’s bolete or powdery sulfur bolete, is an ectomycorrhizal fungus. This species is the type species of the genus *Pulveroboletus* and is distributed in East Asia, Australia, and America. This fungus grows in a mycorrhizal relationship with oak trees (Rodríguez-Ramírez and Moreno 2010). It has long been used in traditional Chinese medicine for the treatment of pain, stiffness, and bleeding. Furthermore, vulpinic acid, atrometic acid, isoxerocomic acid, variegatic acid, xerocomorubin, and variegatorubin have been isolated from the fruit bodies of this fungus (Gill 1994; Kim et al. 2017). To date, over 900 and under 160 mitochondrial genomes of fungi and basidiomycetes, respectively, are available in public databases within the National Center for Biotechnology Information (NCBI). However, only two complete mitochondrial genome sequences from *Boletaceae* (*Tylopilus plumbeoviolaceoides* and *Xerocomus impolitus*) are present in the NCBI repository. In this study, the complete mitochondrial genome of *P. ravenelii* was sequenced using the next-generation sequencing technology, and the phylogenetic relationships within Boletales were analyzed.

A specimen of *P. ravenelii* was collected from Jeongseon-gun, Gangwon Province, South Korea (128°35′25″E, 37°28′26″N). This collection area is not a protected area. A voucher herbarium specimen (accession number: KA20-0520) was deposited at the herbarium of the Korea National Arboretum (https://kna.forest.go.kr/kfsweb/kfs/subIdx/Index.do?mn=UKNA), Korea. This collection was identified by CS Kim (contact CSK, email:changsun84@korea.kr). This study has been granted an exemption by the ethics committee of Korea National Arboretum. Specific permission is not needed, because no relevant animals were involved. This article was conducted in compliance with the regulations of the Korea National Arboretum.

Genomic DNA was isolated from the basidiocarp and used to construct an Illumina paired-end (PE) library per the manufacturer’s protocol. The library was sequenced using Illumina PE sequencing at the PHYZEN Genomics Institute (PHYZEN Co., Seongnam, South Korea). High-quality PE reads obtained after trimming were assembled *de novo* using the CLC genome assembler (v. 4.21, CLC Inc., Aarhus, Denmark). The initially assembled contigs derived from mitochondrial genome sequences were further processed to generate a single draft sequence, as previously documented (Lee et al. 2018). The draft sequence was manually corrected and gap-filled using a series of PE read mappings. The complete mitochondrial genome sequence was annotated using GeSeq (https://chlorobox.mpimp-golm.mpg.de/geseq-app.html) and manually curated using the Artemis annotation tool (Rutherford et al. 2000) with NCBI BLASTN search.

The complete mitochondrial genome sequence of *P. ravenelii* (GenBank accession no. OM405130) was found to be 43,528 bp in length, which is the largest among the previously reported *Boletaceae* mitogenomes. The complete mitogenome comprised 15 protein-coding genes (*atp6*, *atp8*, *atp9*, *cob*, *cox1*, *cox2*, *cox3*, *nad1*, *nad2*, *nad3*, *nad4*, *nad4L*, *nad5*, *nad6*, and *rps3*), 15 transfer RNA genes, two ribosomal RNA genes, and eight open reading frames (ORFs). Its base composition was A (37.52%), C (11.14%), G (12.21%), and T (39.13%), with an overall GC content of 23.35%. Start-codon (*rps3* and *nad3* genes) was not found because there are often genes in organelles that do not start with methionine. The termination codon of them is TAA.

Phylogenetic analysis was performed based on multiple alignments of protein-coding sequences in the mitochondrial genomes using MEGA7 (Kumar et al. 2016). The results revealed that *Xerocomus impolitus* (GenBank accession no. NC_056808) was positioned in a sister clade. In this study, the phylogenetic position of the genus *Pulveroboletus* was determined for the first time (Figure 1). This novel information on the complete mitochondrial genome sequence of *P. ravenelii* would improve the understanding of the evolution of this species and its phylogenetic relationships with the related taxa.

**Figure 1.**
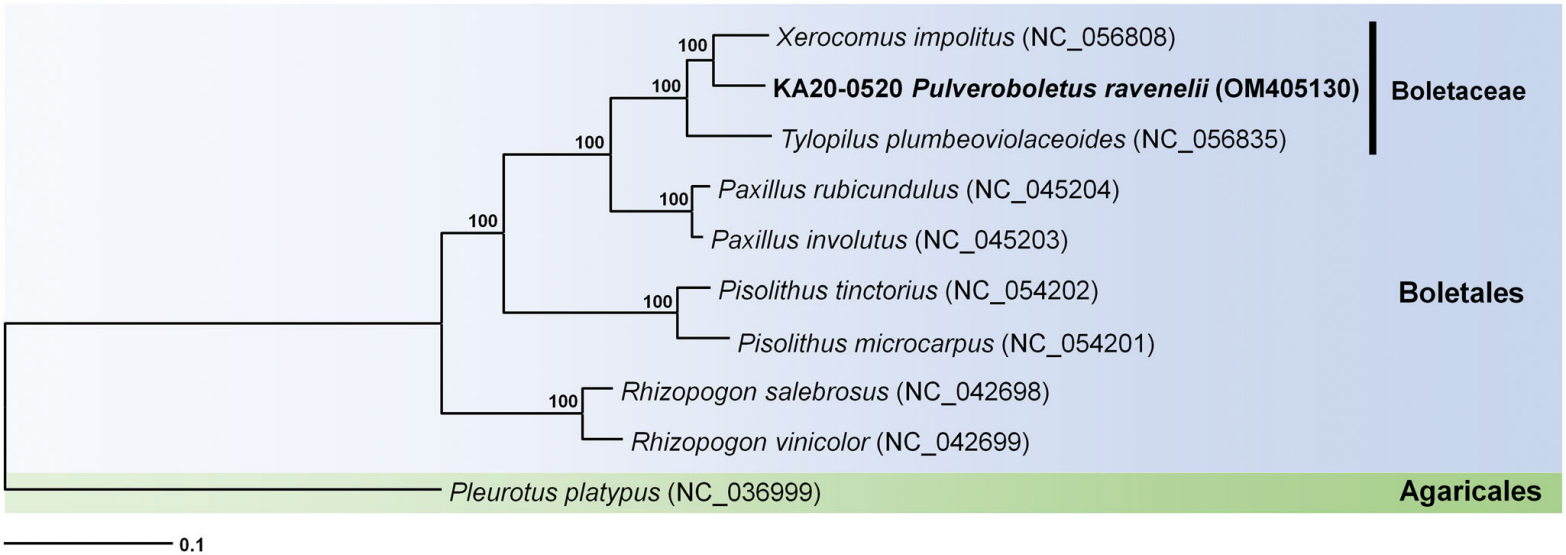
Phylogenetic tree of *Pulveroboletus ravenelii* and eight related taxa. Sequences of protein-coding regions in the mitochondrial genome were aligned using MAFFT and MEGA 7.0. Numbers in the nodes indicate bootstrap support values (>80%) from 1000 replicates.

## Data Availability

The genome sequence data that support the findings of this study are openly available in the GenBank of NCBI at https://www.ncbi.nlm.nih.gov/ under the accession no. OM405130. The associated BioProject, SRA, and Bio-Sample numbers are PRJNA801009, SRR17775244, and SAMN25289627, respectively.
